# Ethanol Extract of *Aurantiochytrium mangrovei* 18W-13a Strain Possesses Anti-inflammatory Effects on Murine Macrophage RAW264 Cells

**DOI:** 10.3389/fphys.2018.01205

**Published:** 2018-09-26

**Authors:** Shinya Takahashi, Midori Sakamaki, Farhana Ferdousi, Masaki Yoshida, Mikihide Demura, Makoto M. Watanabe, Hiroko Isoda

**Affiliations:** ^1^Faculty of Life and Environmental Sciences, University of Tsukuba, Tsukuba, Japan; ^2^Alliance for Research on the Mediterranean and North Africa, University of Tsukuba, Tsukuba, Japan; ^3^Algae Biomass and Energy System R&D Center, University of Tsukuba, Tsukuba, Japan

**Keywords:** anti-inflammation, *Aurantiochytrium*, microalgae, RAW264 cells, pro-inflammatory cytokines, nitric oxide, lipopolysaccharide

## Abstract

In this study, the effects of an ethanolic extract of *Aurantiochytrium mangrovei* 18W-13a strain (AM18W-13a) on lipopolysaccharide (LPS)-induced inflammatory responses in RAW264 murine macrophages were studied. Pre-treatment with the AM18W-13a extract significantly suppressed the LPS-induced production of nitric oxide and pro-inflammatory cytokines. RAW264 cells treated with the AM18W-13a extract for 1 and 24 h were subjected to DNA microarray analyses for detecting the differentially expressed genes. The treatment of RAW264 cells with the AM18W-13a extract for 24 h significantly suppressed the expression of several genes associated with inflammation or chemotaxis. Furthermore, treatment with the AM18W-13a extract for 1 h suppressed the expression of *Pde4b*, but induced the expression of *Egr2* and *Egr3* in RAW264 cells. Additionally, the AM18W-13a extract significantly enhanced the expression of certain anti-inflammatory mediators. This study is the first report of the anti-inflammatory effects of the AM18W-13a extract and its mechanism of action in LPS-stimulated murine macrophages.

## Introduction

Inflammation is a highly regulated immune response, in which tissues respond to injury and infection for eliminating the cause of injury, repairing damages, and returning to the original healthy state (Morson, 1970). However, inflammatory dysregulation can lead to chronic inflammation, which is now recognized as the cause of a wide range of diseases and disorders, including autoimmune disorders, neurodegenerative disorders, metabolic syndrome, cardiovascular diseases, and even cancers (Medzhitov, 2010; Murakami and Hirano, 2012). The major classes of drugs used to suppress inflammation are non-steroidal anti-inflammatory drugs and corticosteroids. However, their use is limited owing to several undesirable side effects, including peptic ulceration, osteoporosis, high blood pressure, and kidney problems (Sostres et al., 2010). Natural product-derived complementary medicines have recently gained considerable attention as better alternatives to non-steroidal anti-inflammatory drugs and corticosteroids for treating inflammation, owing to their therapeutic activities. Algae, especially microalgae, are considered to be a promising source of novel bioactive natural compounds that can serve as raw materials for functional foods, cosmetic products, and drug discovery. Several studies have reported the anti-inflammatory effects of microalgal extracts on mammalian cells (Soontornchaiboon et al., 2012; Robertson et al., 2015; Sibi and Rabina, 2016). However, microalgal species produce several other potent and biologically active novel compounds, which require further exploration.

The 18W-13a strain of *Aurantiochytrium mangrovei* (AM18W-13a; previously known as *Aurantiochytrium* sp. 18W-13a) is a microalgal strain, which has been recently discovered and isolated from a mangrove area in the Okinawa Prefecture of Japan (Kaya et al., 2011). It has a very high efficiency of hydrocarbon production, including the production of the hydrocarbon squalene (Nakazawa et al., 2012). The AM18W-13a strain belongs to the genus *Labyrinthula*, which has been reported to contain several bioactive substances, including squalene, astaxanthin, and canthaxanthin (Christaki et al., 2013). It is therefore possible that the AM18W-13a strain is a potential source of numerous biologically active compounds.

At present, one of the potential approaches employed for screening anti-inflammatory drugs involves the inhibition of pro-inflammatory cytokines and the production of nitric oxide (NO) in lipopolysaccharide (LPS)-stimulated macrophages. This study is the first attempt to evaluate the anti-inflammatory activity of an ethanolic extract of the AM18W-13a strain in LPS-stimulated murine RAW264 cells.

## Materials and Methods

### Preparation of the AM18W-13a Extract

The AM18W-13a strain was provided by the Algae Biomass and Energy System R&D Center, University of Tsukuba, Japan. The lyophilized powder of the AM18W-13a strain (0.5 g) was extracted with 5 ml of 99.5% ethanol and kept in the dark at room temperature for 2 weeks. After centrifugation at 190 ×*g* for 10 min, the supernatant of the extracted sample was collected and filtered using a 0.22-μm filter unit. The final solution of the extract was then stored in the dark at -80°C until use.

### Culture of RAW264 Cells

RAW264 murine macrophages were purchased from RIKEN BioResource Center (RCB0535, RIKEN BRC, Tsukuba, Japan). The RAW264 cells were cultured in Dulbecco’s modified Eagle medium supplemented with 10% heat-inactivated fetal bovine serum and penicillin-streptomycin solution at 37°C in a humidified incubator containing 5% CO_2_. The cells were seeded in 96-well cell culture plates at a density of 2.0 × 10^5^ cells per well and incubated at 37°C for 24 h.

### Preparation of LPS

LPS (*Escherichia coli* serotype O111:B4) was purchased from EMD Millipore Co. (Billerica, MA, United States). LPS (5 mg) was dissolved in 2 ml of phosphate-buffered saline without divalent cations [PBS (-)], and was stored in the dark at -80°C until use.

### Cell Proliferation Assay

The effects of the AM18W-13a extract on the proliferation of RAW264 cells were determined by the mitochondrial-dependent reduction of 3-(4,5-dimethylthiazol-2-yl)-2,5-diphenyl tetrazolium bromide (MTT) to formazan (Mosmann, 1983). The RAW264 cells were treated with the AM18W-13a extract at concentrations ranging from 1/10000 to 1/100 at 37°C for 24 h, while the cells in the control group were left untreated. Following treatment, the MTT solution was added to each well (10 μl/well) and incubated at 37°C for 4 h. The formazan crystals thus formed were dissolved by the addition of 100 μl of 10% sodium dodecyl sulfate (Wako, Japan) to each well, and incubated overnight at 37°C. After incubation, the absorbance was measured at 570 nm using a microplate reader (Power Scan HT, BioTek Japan Inc.). The absorbance values were normalized to those of the medium and are represented as a percentage of the control (medium).

### Measurement of NO Production

The total concentration of nitrite, which is the metabolic end product of NO metabolism, was measured using the two-step Griess diazotization reaction (Sharma et al., 2007). The RAW264 cells were treated with the AM18W-13a extract at 37°C for 24 h. LPS solution (1 ng/ml) was subsequently added to each well and incubated at 37°C for 12 h. The cell culture supernatant was then mixed with an equal volume of Griess reagent [1% sulfanilic acid and 0.1% *N*-(1-naphthyl) ethylenediamine dihydrochloride in 2.5% phosphoric acid; 1:1]. The absorbance was measured at 540 nm using a microplate reader (Power Scan HT, BioTek Japan Inc.). The concentration of NO was calculated from the standard curve of sodium nitrite.

### Measurement of Inflammatory Cytokines

The cells were treated with the AM18W-13a extract at 37°C for 24 h. LPS solution (1 ng/ml) was subsequently added to each well, and incubated at 37°C for 12 h. The levels of inflammatory cytokines in the cell culture supernatant were measured using the Bio-Plex Pro^TM^ Mouse Cytokine assay kits (Bio-Rad, United States) according to the manufacturer’s instructions. The data were obtained using the MAGPIX xPONENT 4.2 system (Merck Millipore Co., United States). Specific antibodies against tumor necrosis factor (TNF)-α, interleukin (IL)-6, IL-1β, monocyte chemoattractant protein-1 (MCP-1), and granulocyte-macrophage colony-stimulating factor (GM-CSF) were used for this assay.

### DNA Microarray Analysis for Gene Expression Profiling in RAW264 Cells

The RAW264 cells were seeded in 6-well plates at the density of 2.0 × 10^5^ cells/well and incubated at 37°C for 24 h. The cells were subsequently treated with the AM18W-13a extract at 37°C for 1 and 24 h. Total RNA was isolated using ISOGEN (Nippon Gene, Tokyo, Japan), according to the manufacturer’s instructions. The amplified RNA (aRNA) was synthesized using the GeneChip 3′ IVT PLUS Reagent Kit (Thermo Fisher Scientific, United States). Hybridization was achieved using the Affymetrix GeneChip Mouse Genome 430 Array Strip (Thermo Fisher Scientific). The images were obtained by the GeneAtlas^TM^ Imaging Station and analyzed using the GeneAtlas^TM^ Workstation (Thermo Fisher Scientific), according to the manufacturer’s instructions.

The DNA microarrays were classified and analyzed by the gene set enrichment analysis approach, using the Database for Annotation, Visualization and Integrated Discovery (DAVID) server, v6.8 (National Institute of Allergy and Infectious Diseases (NIAID), NIH, United States^1^) (Huang et al., 2007a,b). The genes containing the gene ontologies for inflammatory response, immune response, chemotaxis, and other cellular activities such as cytokine expression, NO signaling, response to LPS, response to wounding, nuclear factor kappa (NFk) B signaling, and Janus kinase/signal transducers and activators of transcription (JAK/STAT) signaling were manually selected from the clustered genes.

### Analysis of the Gene Expression of RAW264 Cells Using Real-Time Reverse Transcription Polymerase Chain Reaction (RT-PCR)

The effects of the AM18W-13a extract on the gene expression of RAW264 cells were evaluated using real-time RT-PCR. The RAW264 cells were seeded in 6-well plates at a density of 2.0 × 10^5^ cells/well and incubated at 37°C for 24 h. RAW264 cells were treated with the AM18W-13a extract at 37°C for 3 h. Total RNA was isolated using ISOGEN (Nippon Gene), according to the manufacturer’s instructions. The complementary DNA (cDNA) was synthesized from 1 μg of total RNA using the SuperScript III reverse transcription kit, according to the manufacturer’s protocol.

TaqMan real-time RT-PCR amplification reactions were carried out for quantifying the transcripts using the Applied Biosystems 7500 Fast Real-Time System (Thermo Fisher Scientific). The primer sets and the TaqMan Universal PCR Master Mix were obtained from Thermo Fisher Scientific. Specific primers for *Gapdh* (Mm99999915_g1), *Atf3* (Mm00476033_m1), *Socs1* (Mm00782550_s1), and *Socs3* (Mm00545913_s1) were used.

### Statistical Analyses

All the results represent the mean of three replicate determinations ± standard deviation (SD). Student’s *t*-test was used for statistical evaluation. Statistical significance was considered at *p* < 0.05, unless otherwise stated.

## Results

### Effects of the AM18W-13a Extract on the Proliferation of RAW264 Cells

**FIGURE 1:**
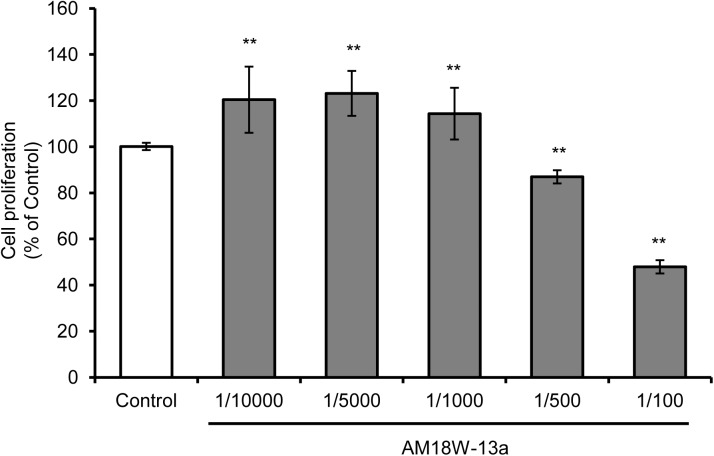
Effects of the AM18W-13a extract on the proliferation of RAW264 cells. The RAW264 cells were treated with the AM18W-13a extract at concentrations of 1/10000, 1/5000, 1/1000, 1/500, and 1/100 for 24 h. The cells in the control group were left untreated. Cell proliferation was measured by the MTT assay after treatment. The values represent the mean ± SD of results obtained from experiments performed in triplicate and are expressed as percentages relative to the results obtained from the control group. The mean value of the experimental result that significantly differed from that of the control group is indicated by asterisks (^∗∗^*p* < 0.01).

The results of the MTT assay demonstrated that treatment with the AM18W-13a extract at concentrations ranging from 1/10000 to 1/1000 significantly increased the proliferation of RAW264 cells by 20%, in comparison to that of the untreated cells (*p* < 0.01). However, treatment with the AM18W-13a extract at concentrations of 1/500 and 1/100 significantly reduced the proliferation of RAW264 cells to 80 and 50%, respectively (**Figure 1**). Therefore, the AM18W-13a extract was used at a dilution of 1/1000 for further experiments.

### Effects of the AM18W-13a Extract on the Proliferation and Production of NO in LPS-Stimulated RAW264 Cells

**FIGURE 2:**
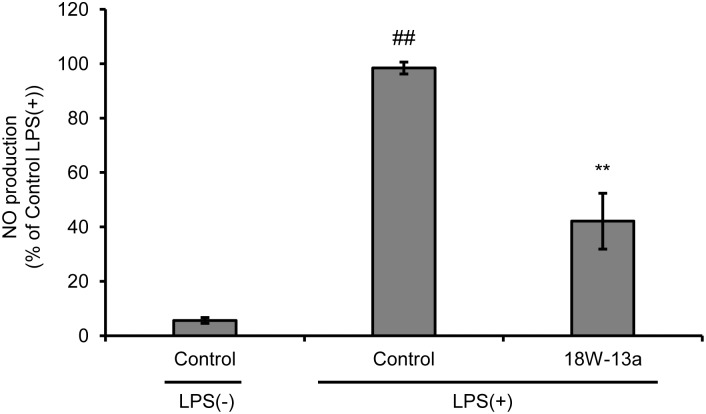
Effects of the AM18W-13a extract (18W-13a) on the LPS-induced production of NO in RAW264 cells. The cells were treated with the AM18W-13a extract at a concentration of 1/1000 for 24 h, while the cells in the control group were left untreated. Following treatment, the cells were activated by incubating with LPS (1 ng/ml) for 12 h. The quantity of NO produced was measured by the Griess reaction. The values represent the mean ± SD of results obtained from experiments performed in triplicate and are expressed as percentages relative to the results obtained from the control LPS (+) group. The mean value of the experimental result that significantly differed from that of the control LPS (–) group is indicated by ^##^*p* < 0.01. The mean value that is significantly different from that of the control LPS (+) group is indicated by asterisks (^∗∗^*p* < 0.01).

We evaluated the effects of LPS on the proliferation of RAW264 cells and the production of NO. We found that the proliferation of LPS (0.01–100 ng/ml)-treated RAW264 cells was significantly reduced (**Supplementary Figure S1**), while the production of NO was significantly enhanced in a dose-dependent manner, following treatment with the extract (**Supplementary Figure S2**). The peak of NO production was observed when the concentration of LPS was 1 ng/ml. The cells were therefore treated with 1 ng/ml LPS in the subsequent experiments performed. Treatment with the AM18W-13a extract at a concentration of 1/1000 for 24 h significantly reduced the LPS-induced production of NO (*p* < 0.01) by approximately 50% (**Figure 2**).

### Effect of the AM18W-13a Extract on the LPS-Induced Production of Pro-Inflammatory Cytokines

**FIGURE 3:**
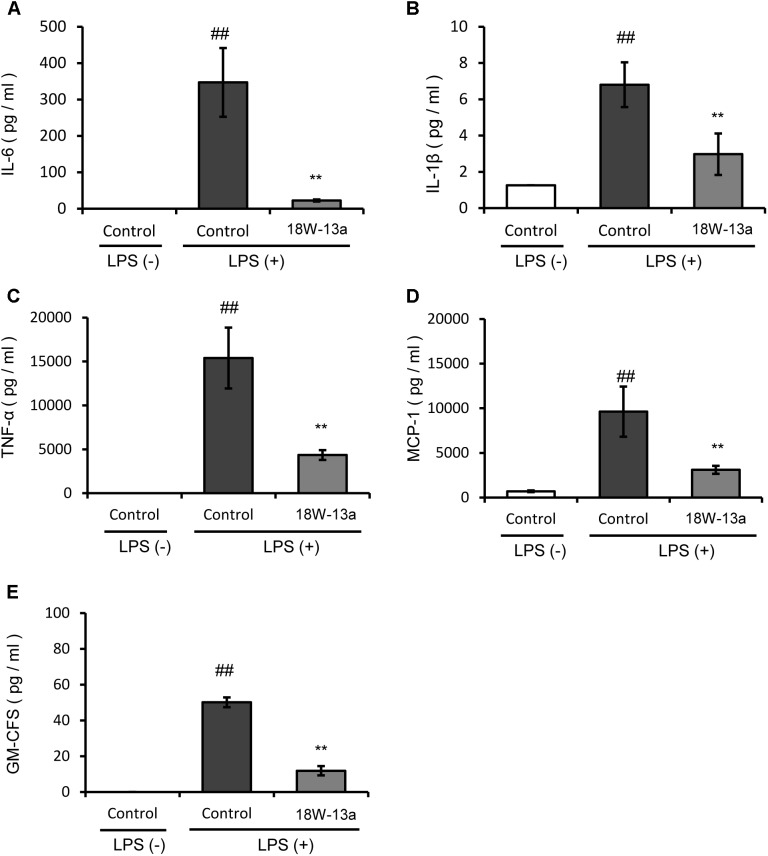
Effects of the AM18W-13a extract (18W-13a) on the LPS-induced expression of pro-inflammatory cytokines in RAW264 cells. The levels of **(A)** IL-6, **(B)** IL-1β, **(C)** TNF-α, **(D)** MCP-1, and **(E)** GM-CSF in RAW264 cells were determined. The cells were treated with the AM18W-13a extract at a concentration of 1/1000 for 24 h, while the cells in the control group were left untreated. Following treatment, the cells were activated by incubation with LPS (1 ng/ml) for 12 h. The pro-inflammatory mediators secreted thereafter were determined by ELISA with the MAGPIX xPONENT system. The values represent the mean ± SD of results obtained from experiments performed in duplicate. The mean value of the experimental result that significantly differed from that of the control LPS (–) group is indicated by ^##^*p* < 0.01. The mean value of the experimental result that significantly differed from that of the control LPS (+) group is indicated by ^∗∗^*p* < 0.01.

The LPS-induced production of cytokines in the RAW264 cells treated with the AM18W-13a extract was compared to that of the untreated cells. **Figure 3** demonstrates that the levels of IL-6, IL-1β, TNF-α, MCP-1, and GM-CSF in the cells treated with the AM18W-13a extract were significantly lower than those of the untreated cells (*p* < 0.01).

### Analysis of Gene Expression in RAW264 Cells Treated With the AM18W-13a Extract

The anti-inflammatory properties and the target genes of the AM18W-13a extract were determined by gene expression profiling using DNA microarray analysis of the RAW264 macrophages treated with the AM18W-13a extract. Three independent biological replicates were selected for each condition in the DNA microarray analysis. The differentially expressed genes were analyzed by gene-enrichment analysis, using the web-based tool, DAVID. One hour after treatment with the AM18W-13a extract at a concentration of 1/1000, the expression of six genes was upregulated, while that of nine genes was downregulated (fold change >1.4 and <1.4; **Table 1**). The expression of genes encoding transcriptional regulators and zinc-finger proteins were significantly affected (**Supplementary Table S1**). In particular, the expression of genes encoding the zinc finger transcription factors, such as early growth response 2 (*Egr2)* and *Egr3*, increased following treatment with the extract, while the expression of phosphodiesterase 4B (*Pde4b*) decreased after treatment with the AM18W-13a extract.

**Table 1:** Gene clusters whose expression was more than 1.4 times higher or lower than that in the control group, following treatment with the AM18W-13a extract for 1 h.

Upregulated genes			
Gene symbol	Gene name	Ratio	
		1 h	24 h
*Vapb*	Vesicle-associated membrane protein, associated protein B and C	1.88	1.28
*Rgs1*	Regulator of G-protein signaling 1	1.70	0.51
*Egr3*	Early growth response 3	1.53	1.09
*Naa35*	N(alpha)-acetyltransferase 35, NatC auxiliary subunit	1.56	0.90
*Egr2*	Early growth response 2	1.55	1.05
*Zfp146*	Zinc finger protein 146	1.49	1.02

**Downregulated genes**			
**Gene symbol**	**Gene title**	**Ratio**	
		**1 h**	**24 h**

*Ndufs8*	NADH dehydrogenase (ubiquinone) Fe-S protein 8	0.51	1.02
*Cxcl2*	Chemokine (C-X-C motif) ligand 2	0.52	0.58
*Nfkbiz*	Nuclear factor of kappa light polypeptide gene enhancer in B cells inhibitor, ze	0.60	1.12
*Ppm1b*	Protein phosphatase 1B, magnesium dependent, beta isoform	0.61	0.87
*Hnrnpab*	Heterogeneous nuclear ribonucleoprotein A/B	0.62	1.29
*Pde4b*	Phosphodiesterase 4B, cAMP specific	0.66	1.15
*Traf1*	TNF receptor-associated factor 1	0.68	0.94
*Nfkbie*	Nuclear factor of kappa light polypeptide gene enhancer in B cells inhibitor, ep	0.69	1.04
*Ier3*	Immediate early response 3	0.70	0.64

*The values indicate the average of results obtained from independent experiments performed in duplicate or triplicate*.

Treatment with the AM18W-13a extract at a concentration of 1/1000 for 24 h upregulated the expression of 102 genes and downregulated the expression of 88 genes (fold change >1.5 and <1.5; **Supplementary Tables S2**, **S3**). We used the DAVID gene ontology tool for identifying the genes related to inflammatory responses in the RAW264 cells that had been treated with the AM18W-13a extract. The genes were classified into 35 functionally annotated clusters, out of which six were selected that contained the following terms: chemotaxis, inflammatory, immune response, cytokine-cytokine receptor interaction, and chemokine activity. Finally, 24 genes with downregulated expression profiles and 22 genes with upregulated expression profiles were selected (**Tables 2**, **3** and **Supplementary Tables S4**, **S5**). The expression of genes encoding inflammatory substances, including C-C motif chemokine ligand 3 (*Ccl3*), *Ccl4, Tnf*, and prostaglandin-endoperoxide synthase 2 (*Ptgs2*), decreased after treatment with the AM18W-13a extract. Additionally, the expression of genes encoding proteins involved in inflammatory responses or chemotaxis, including ATP-binding cassette subfamily c member 1 (*Abcc1*), cluster of differentiation 36 (*Cd36*), fms-related tyrosine kinase 1 (*Flt1*), S100 calcium binding protein a8 (*S100a8*), and serpin family E member 1 (*Serpine1*) decreased following treatment with the extract (**Table 2** and **Supplementary Table S4**). However, the expression of certain genes involved in the anti-inflammatory pathways, including sphingosine-1-phosphate receptor 1 (*S1pr1*), IL 10 receptor subunit alpha (*IL10ra*), and TSC22 domain family member 3 (*Tsc22d3*), increased after treatment with the AM18W-13a extract (**Table 3** and **Supplementary Table S5**).

**Table 2:** Genes whose expression was more than 1.5 times lower than that in the control group, following treatment with the AM18W-13a extract for 24 h, were selected from the gene clusters.

Gene symbol	Gene name	Ratio	
		1 h	24 h
*Trem3*	Triggering receptor expressed on myeloid cells 3	0.97	0.57
*Tnf*	Tumor necrosis factor	0.68	0.56
*Abcc1*	ATP-binding cassette, subfamily C (CFTR/MRP), member 1	1.08	0.54
*Flt1*	FMS-like tyrosine kinase 1	1.03	0.54
*Gch1*	GTP cyclohydrolase 1	1.02	0.54
*Pdgfb*	Platelet derived growth factor, B polypeptide	1.12	0.53
*Plau*	Plasminogen activator, urokinase	0.99	0.53
*S100a8*	S100 calcium binding protein A8 (calgranulin A)	1.06	0.53
*Prkar2b*	Protein kinase, cAMP dependent regulatory, type II beta	1.02	0.52
*Slpi*	Secretory leukocyte peptidase inhibitor	1.01	0.52
*Irg1*	Immunoresponsive gene 1	0.94	0.48
*Serpinb9*	Serine (or cysteine) peptidase inhibitor, clade B, member 9	1.03	0.47
*Slc11a1*	Solute carrier family 11 (proton-coupled divalent metal ion transporters), member	0.90	0.47
*Dcstamp*	Dendrocyte expressed seven transmembrane protein	1.05	0.46
*Gstp1*	Glutathione S-transferase, pi 1	1.01	0.46
*Nrp1*	Neuropilin 1	0.98	0.45
*Ptgir*	Prostaglandin I receptor (IP)	0.91	0.44
*Serpine1*	Serine (or cysteine) peptidase inhibitor, clade E, member 1	1.14	0.44
*Ccl4*	Chemokine (C-C motif) ligand 4	1.12	0.43
*Clec4n*	C-type lectin domain family 4, member n	0.92	0.41
*Cd36*	CD36 antigen	0.98	0.41
*Ptgs2*	Prostaglandin-endoperoxide synthase 2	1.12	0.39
*Ccl3*	Chemokine (C-C motif) ligand 3	1.12	0.34
*Hmox1*	Heme oxygenase (decycling) 1	1.34	0.31

*The values indicate the average of results obtained from independent experiments performed in duplicate or triplicate*.

**Table 3:** Genes whose expression was 1.5 times higher than that in the control group, following treatment with the AM18W-13a extract for 24 h, were selected from the gene clusters.

Gene symbol	Gene name	Ratio	
		1 h	24 h
*St6gal1*	Beta galactoside alpha 2,6 sialyltransferase 1	1.02	2.35
*S1pr1*	Sphingosine-1-phosphate receptor 1	1.02	2.12
*Fcgr3*	Fc receptor, IgG, low affinity III	0.90	2.07
*Lgals9*	Lectin, galactose binding, soluble 9	1.02	2.03
*Clec7a*	C-type lectin domain family 7, member a	1.02	2.00
*Cxcr4*	Chemokine (C-X-C motif) receptor 4	0.95	1.99
*Blnk*	B cell linker	0.99	1.87
*Cd28*	CD28 antigen	0.93	1.86
*Tgfbr1*	Transforming growth factor, beta receptor I	1.08	1.84
*Ifi44l*	Interferon-induced protein 44	1.00	1.82
*Il10ra*	Interleukin 10 receptor, alpha	0.79	1.78
*Lyst*	Lysosomal trafficking regulator	0.97	1.77
*Tifab*	TRAF-interacting protein with forkhead-associated domain, family member B	0.97	1.76
*Tsc22d3*	TSC22 domain family, member 3	1.02	1.75
*Gas6*	Growth arrest specific 6	0.93	1.74
*Cxcl16*	Chemokine (C-X-C motif) ligand 16	1.00	1.72
*C3ar1*	Complement component 3a receptor 1	1.00	1.70
*Aif1*	Allograft inflammatory factor 1	0.92	1.66
*Irf9*	Interferon regulatory factor 9	1.03	1.63
*C1qb*	Complement component 1, q subcomponent, beta polypeptide	1.03	1.60
*Il18*	Interleukin 18	1.01	1.52
*Il6ra*	Interleukin 6 receptor, alpha	1.07	1.56

*The values indicate the average of results obtained from independent experiments performed in duplicate or triplicate*.

### Effects of the AM18W-13a Extract on the Expression of Anti-inflammatory Mediators

**FIGURE 4:**
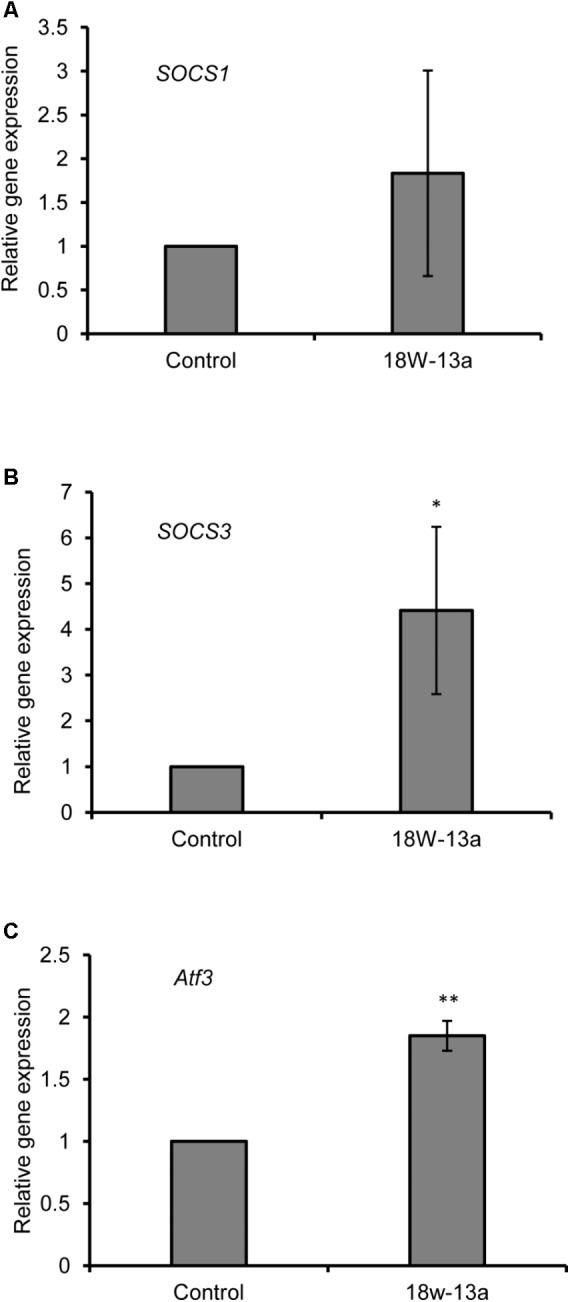
Effects of the AM18W-13a extract (18W-13a) on the expression of *Socs1, Socs3*, and *Atf3* genes in RAW264 cells. The RAW264 cells were treated with the AM18W-13a extract at a concentration of 1/1000 for 3 h, while the cells in the control group were left untreated. Following treatment, the expression of **(A)** *Socs1*, **(B)** *Socs3*, and **(C)** *Atf3* was evaluated by real-time RT-PCR. The values represent the mean ± SD of results obtained from experiments performed in triplicate. The mean value of the experimental result that significantly differed from those of the control group is indicated by asterisks (^∗^*p* < 0.05 and ^∗∗^*p* < 0.01).

The expression of certain genes that negatively regulate the inflammatory process in the RAW264 cells treated with the AM18W-13a extract was evaluated. Treatment with the AM18W-13a extract at a concentration of 1/1000 for 3 h significantly increased the expression of *Socs3* (*p* < 0.05) and *Atf3* (*p* < 0.01) by 4.41- and 1.84-fold, respectively, compared to the control group. Additionally, the expression of *Socs1* tended to increase after treatment with the AM18W-13a extract (**Figure 4**).

The interactions of the anti-inflammatory regulators and the two zinc finger-type transcription factors, *Egr2* and *Egr3*, were investigated using the GeneMANIA server^2^, which can predict several gene interaction networks from a gene list (Warde-Farley et al., 2010). Among *Atf3, Egr2, Egr3, Socs1*, and *Socs3*, the coexpression of *Egr2* with *Egr3, Atf3*, and *Socs3* and the coexpression of *Egr3* with *Egr2, Atf3*, and *Socs1* were confirmed (**Supplementary Figure S3**).

## Discussion

In this study, we evaluated the anti-inflammatory effects of the AM18W-13a extract on LPS-induced responses in murine RAW264 cells. Macrophages play key roles in all stages of the inflammatory response by acquiring distinct functional phenotypes that are directed by the tissue type and environmental cues (Mosser and Edwards, 2008). LPS is a potent activator of macrophages that triggers the abundant secretion of pro-inflammatory cytokines and the production of NO, which is an inflammatory mediator. The results of this study indicated that the AM18W-13a extract could potentially inhibit the LPS-induced production of NO and the secretion of pro-inflammatory cytokines, including IL-6, IL-1β, TNF-α, MCP-1, and GM-CFS, in RAW264 cells. Moreover, the expression of certain inflammatory substances, including *Ccl3, Ccl4, Tnf*, and *Ptgs2*, was substantially attenuated by the AM18W-13a extract in LPS-stimulated macrophages. Since these genes were related to ‘chemotaxis’ and ‘inflammation,’ it can be assumed that the AM18W-13a extract had anti-inflammatory effects on RAW264 cells.

The treatment of RAW264 cells with the AM18W-13a extract significantly enhanced the expression of *Socs3* and *Atf3*. The expression of *Socs1* also tended to increase following treatment with the extract. The majority of SOCS proteins act in a classical negative-feedback loop for inhibiting cytokine signal transduction. Both the SOCS1 and SOCS3 proteins directly inhibit the tyrosine kinase activity of JAKs and act as negative regulators of the cytokine-induced JAK/STAT pathway. It has also been suggested that SOCS proteins are important negative regulators of toll-like receptor (TLR) signaling. SOCS3 inhibits the IL-1-induced transcription and activation of signaling pathways mediated by NF-κB and JNK/p38 by inhibiting the association between TNF receptor-associated factor 6 and TGF β-activated kinase 1 (Frobose et al., 2006). Additionally, SOCS3 strongly inhibits the IL-6-induced activation of STAT3 but does not affect the IL-10-induced activation of STAT3. Therefore, the sustained activation of STAT3 inhibits the TLR-induced production of cytokines (Yoshimura et al., 2012). However, SOCS1 binds to the p65 subunit of NF-κB and induces its degradation, thus subsequently inhibiting the NF-κB pathway (Strebovsky et al., 2011). The lack of SOCS1 in macrophages makes them hypersensitive to stimuli, such as LPS, resulting in the increased production of pro-inflammatory cytokines (Strebovsky et al., 2011). ATF3 is a member of the ATF/cyclic adenosine monophosphate (cAMP) response element-binding protein of the basic leucine zipper transcription factors. ATF is reported to be an inducible negative regulator of TLR4 signaling (Gilchrist et al., 2006). A previous study reported that the expression of genes encoding pro-inflammatory cytokines (IL-6 and IL-12b) increases in *Atf3*-deficient mice, indicating that ATF3 negatively regulates the expression of genes related to the inflammatory response (Gilchrist et al., 2008). Additionally, the expression of pro-inflammatory cytokines is significantly reduced in the macrophages of congenic *Atf3*^+/+^ mice under both LPS-stimulated and unstimulated conditions (Khuu et al., 2007). ATF3 also inhibits the NF-κB signaling pathway by binding either to the promoter of the NF-κB-dependent genes (Gilchrist et al., 2006) or by directly binding to the p65 subunit of NF-κB (Kwon et al., 2015). Altogether, our findings suggest that the anti-inflammatory effects of the AM18W-13a extract could be related to the increased expression of *Atf3, Socs1*, and *Socs3*.

Furthermore, the results of DNA microarray analysis revealed that treatment with the AM18W-13a extract for 1 h suppressed the expression of *Pde4b* and enhanced the expression of *Egr2* and *Egr3* in RAW264 cells. PDE4B is a member of the PDE enzyme superfamily, which hydrolyzes cAMP to the inactive 5′ monophosphatase. Since cAMP exhibits anti-inflammatory properties in a variety of cell types (Oldenburger et al., 2012), the degradation of cAMP by PDE promotes the pathogenesis of inflammatory diseases. *Egr2* and *Egr3* are well-known homeostasis-related genes that encode proteins of the EGR family, which are zinc finger transcription factors. EGRs play an important role in the development of systemic autoimmune diseases, and the deficiency of EGR2 and EGR3, in particular, are related to the development of chronic inflammatory diseases, such as lupus, in murine models (Gomez-Martin et al., 2010). Recent studies have demonstrated that EGR2 and EGR3 induce the expression of anti-inflammatory regulators, including SOCS1 and SOCS3 (Li et al., 2012; Sumitomo et al., 2013). Additionally, lymphocytes deficient in *Egr2* and *Egr3* have been reported to produce high levels of inflammatory cytokines (Li et al., 2012). Interestingly, the expression of EGR2 is induced when the intracellular levels of cAMP are elevated (Schmid et al., 2014). We can therefore hypothesize that the AM18W-13a extract inhibits the intracellular degradation of cAMP by regulating the expression of the *Pde4b* gene, and the resulting increase in levels of intracellular cAMP induces the expression of *Egr2* and *Egr3*, which subsequently enhances the expression of *Socs1* and *Socs3*. This mechanism of post-transcriptional regulation has not been proven by direct experimental evidence, but by network-level conservation for confirming the coexpression of *Atf3, Egr2, Egr3, Socs1*, and *Socs3*. It was found that *Egr2* is coexpressed with *Egr3, Atf3*, and *Socs3*, while *Egr3* is coexpressed with *Egr2, Atf3*, and *Socs1*.

**FIGURE 5:**
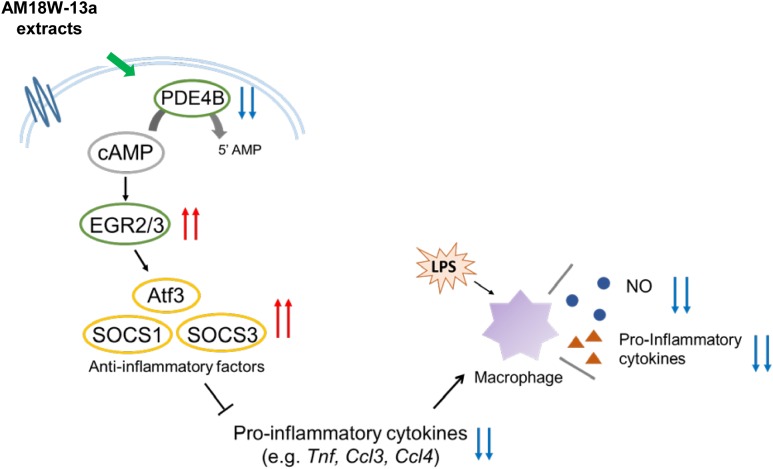
Prediction of the anti-inflammatory effects of the AM18W-13a extract in RAW264 cells. The AM18W-13a extract inhibits the production of LPS-induced inflammatory mediators by increasing the expression of anti-inflammatory mediators such as ATF3, SOCS1, and SOCS3.

In summary, the study reports that the AM18W-13a extract may inhibit the production of NO and the secretion of pro-inflammatory cytokines by upregulating the expression of anti-inflammatory mediators, including ATF3, SOCS1, and SOCS3, by inducing the expression of the transcription factors EGR2 and EGR3, via cAMP (**Figure 5**). These results indicate that the AM18W-13a extract has anti-inflammatory effects on murine macrophages.

## Author Contributions

ST, MS, and HI conceived and designed the experiments. MS performed the experiments. ST, MS, and FF prepared the figures, tables, and manuscript. ST and MS analyzed and interpreted the results. MY, MD, and MW provided the AM18W-13a strain. HI edited and revised the manuscript.

## Conflict of Interest Statement

The authors declare that the research was conducted in the absence of any commercial or financial relationships that could be construed as a potential conflict of interest.

## Footnotes

1 https://david.ncifcrf.gov/

2 http://genemania.org
