# Supplementary material for: Ethanol Extract of Aurantiochytrium mangrovei 18W-13a Strain Possesses Anti-inflammatory Effects on Murine Macrophage RAW264 Cells
Source: Front Physiol. 2018 Sep 26;9:1205. doi: 10.3389/fphys.2018.01205 (PMC6168648; doi:10.3389/fphys.2018.01205)
Supplement: Supplementary file 1 [file Table_1.pdf]

Supplementary table 1. Annotation of genes whose expression was more than 1.4 times higher or lower than that in the control group, following treatment with the AM18W-13a extract for 1 h.

Gene Symbol	Gene Name	Gene Ontology
Egr2	early growth response 2	regulation of transcription, Zinc finger
Egr3	early growth response 3	endothelial cell chemotaxis, regulation of transcription, Zinc finger
Ier3	immediate early response 3	negative regulation of inflammatory response
Cxcl2	chemokine (C-X-C motif) ligand 2	Chemotaxis, inflammatory response
Nfkbiz	nuclear factor of kappa light polypeptide gene enhancer in B cells inhibitor, zeta	inflammatory response, Zinc finger
Nfkbie	nuclear factor of kappa light polypeptide gene enhancer in B cells inhibitor, epsilon	T cell receptor signaling pathway
Ppm1b	protein phosphatase 1B, magnesium dependent, beta isoform	negative regulation of NF-kappaB import into nucleus, Zinc finger

(Continued)

Gene Symbol	Gene Title	Gene Ontology
Traf1	TNF receptor-associated factor 1	positive regulation of NF-kappaB, transcription factor activity, Zinc finger
Pde4b	phosphodiesterase 4B, cAMP specific	neutrophil chemotaxis, cellular responses to lipopolysaccharide,<br>Zinc finger
Vapb	vesicle-associated membrane protein, associated protein B and C	ER to Golgi vesicle-mediated transport
Naa35	N(alpha)-acetyltransferase 35, NatC auxiliary subunit	negative regulation of apoptotic process,
Zfp146	zinc finger protein 146	Transcription regulation, Zinc finger
Hnrnpab	heterogeneous nuclear ribonucleoprotein A/B	positive regulation of transcription,<br>Zinc finger
Ndufs8	NADH dehydrogenase (ubiquinone) Fe-S protein 8	mitochondrial respiratory chain complex I assembly, response to oxidative stress,<br>Zinc finger
Rgs1	regulator of G-protein signaling 1	positive regulation of GTPase activity
