# Supplementary material for: Ethanol Extract of Aurantiochytrium mangrovei 18W-13a Strain Possesses Anti-inflammatory Effects on Murine Macrophage RAW264 Cells
Source: Front Physiol. 2018 Sep 26;9:1205. doi: 10.3389/fphys.2018.01205 (PMC6168648; doi:10.3389/fphys.2018.01205)
Supplement: Supplementary file 2 [file Table_2.pdf]

Supplementary table 2. Genes whose expression was 1.5 times higher than that in the expression in the control group, following treatment with the AM18W-13a extract for 24 h.

Gene Symbol	Gene Name	Ratio	
		1h	24h
Npc2	Niemann Pick type C2	0.89	2.41
Ago4	argonaute RISC catalytic subunit 4	0.99	1.87
St6gal1	beta galactoside alpha 2,6 sialyltransferase 1	1.02	2.35
Dbp	D site albumin promoter binding protein	0.97	1.97
Cxcr4	chemokine (C-X-C motif) receptor 4	0.95	1.99
Gnpda1	glucosamine-6-phosphate deaminase 1 pseudogene /// glucosamine-6-phosphate deami	0.87	2.05
Apoc2	apolipoprotein C-II	0.91	1.94
Tsc22d1	TSC22 domain family, member 1	0.95	1.84
Trp53inp1	transformation related protein 53 inducible nuclear protein 1	1.03	2.61
Pdcd4	programmed cell death 4	1.01	2.47
Lgals9	lectin, galactose binding, soluble 9	1.02	2.03
Cxcl16	chemokine (C-X-C motif) ligand 16	1.00	1.72
Usp18	ubiquitin specific peptidase 18	1.06	2.26
Rnasel	ribonuclease L (2', 5'-oligoisoadenylate synthetase-dependent)	1.09	2.30
Hmgcs1	3-hydroxy-3-methylglutaryl-Coenzyme A synthase 1	0.95	1.68

(Continued)

Gene Symbol	Gene Name	Ratio	
		1h	24h
Pnpla7	patatin-like phospholipase domain containing 7	0.93	1.95
Hpgd	hydroxyprostaglandin dehydrogenase 15 (NAD)	0.91	2.17
Sesn1	sestrin 1	0.92	1.76
Cyp4v3	cytochrome P450, family 4, subfamily v, polypeptide 3	0.97	1.88
Mxd4	Max dimerization protein 4	1.07	2.12
Cd33	CD33 antigen	0.93	2.11
Ms4a6c	membrane-spanning 4-domains, subfamily A, member 6C	1.00	1.90
Slc5a3	solute carrier family 5 (inositol transporters), member 3	1.02	1.69
Fcgr3	Fc receptor, IgG, low affinity III	0.90	2.07
Ifi27l2a	interferon, alpha-inducible protein 27 like 2A	1.07	2.09
Deptor	DEP domain containing MTOR-interacting protein	0.96	1.73
Cd28	CD28 antigen	0.93	1.86
Clec7a	C-type lectin domain family 7, member a	1.02	2.00
Aph1b	anterior pharynx defective 1b homolog (C. elegans)	0.84	1.97
Ptplad2	protein tyrosine phosphatase-like A domain containing 2	0.93	1.98
Wfdc17	extracellular peptidase inhibitor-like /// WAP four-disulfide core domain 17	1.02	1.83
Ranbp3l	RAN binding protein 3-like	1.08	1.82
Rab11fip5	RAB11 family interacting protein 5 (class I)	0.97	1.72

(Continued)

Gene Symbol	Gene Name	Ratio	
		1h	24h
Plxnc1	plexin C1	1.03	1.84
Ypel2	yippee-like 2 (Drosophila)	0.91	1.78
Ms4a6b	membrane-spanning 4-domains, subfamily A, member 6B	0.95	1.88
Lyst	lysosomal trafficking regulator	0.97	1.77
Mafb	v-maf musculoaponeurotic fibrosarcoma oncogene family, protein B (avian)	1.07	1.93
Klhl24	kelch-like 24	1.00	2.02
BB163080	expressed sequence BB163080	0.94	1.95
Tgfbr1	transforming growth factor, beta receptor I	1.08	1.84
Atp6v0d2	ATPase, H+ transporting, lysosomal V0 subunit D2	0.94	1.87
S1pr1	sphingosine-1-phosphate receptor 1	1.02	2.12
Scel	sciellin	0.91	1.84
Blnk	B cell linker	0.99	1.87
Tcp11l2	t-complex 11 (mouse) like 2	0.98	2.05
Id3	inhibitor of DNA binding 3	0.99	1.79
Bbip1	BBSome interacting protein 1	1.03	2.07
Rnf144b	ring finger protein 144B	0.99	1.65

(Continued)

Gene Symbol	Gene Name	Ratio	
		1h	24h
Btg2	B cell translocation gene 2, anti-proliferative	0.94	1.69
Il10ra	interleukin 10 receptor, alpha	0.79	1.78
Tsc22d3	TSC22 domain family, member 3	1.02	1.75
Hebp1	heme binding protein 1	1.01	1.75
Rnase4	ribonuclease, RNase A family 4	1.02	1.75
Rnf180	ring finger protein 180	0.92	1.73
Gas6	growth arrest specific 6	0.93	1.74
Apobec1	apolipoprotein B mRNA editing enzyme, catalytic polypeptide 1	1.04	1.69
Tifab	TRAF-interacting protein with forkhead-associated domain, family member B	0.97	1.76
Ltc4s	leukotriene C4 synthase	1.01	1.84
Fam214a	family with sequence similarity 214, member A	0.96	1.78
Ctsf	cathepsin F	1.00	1.69
Ifi204	interferon activated gene 204	0.98	1.73
Pik3ip1	phosphoinositide-3-kinase interacting protein 1	0.99	1.62
C3ar1	complement component 3a receptor 1	1.00	1.70
Ifi44	interferon-induced protein 44	1.00	1.82

(Continued)

Gene Symbol	Gene Name	Ratio	
		1h	24h
Arl4c	ADP-ribosylation factor-like 4C	0.99	1.64
Zbtb4	zinc finger and BTB domain containing 4	0.99	1.62
Ipo11	importin 11	1.03	1.71
Epsti1	epithelial stromal interaction 1 (breast)	1.01	1.64
Irf9	interferon regulatory factor 9	1.03	1.63
Mndal	myeloid nuclear differentiation antigen like	1.01	1.59
Crebrf	CREB3 regulatory factor	0.93	1.65
Rnf166	ring finger protein 166	1.02	1.58
Aif1	allograft inflammatory factor 1	0.92	1.66
Aldoc	aldolase C, fructose-bisphosphate	1.01	1.60
Bmf	BCL2 modifying factor	1.01	1.60
Slfn8	schlafen 8	0.99	1.61
Il6ra	interleukin 6 receptor, alpha	1.07	1.56
Rmnd5a	required for meiotic nuclear division 5 homolog A (S. cerevisiae)	0.97	1.58
Malat1	metastasis associated lung adenocarcinoma transcript 1 (non-coding RNA)	1.23	1.56
Ap1s2	adaptor-related protein complex 1, sigma 2 subunit	0.95	1.55

(Continued)

Gene Symbol	Gene Name	Ratio	
		1h	24h
Ifi203	interferon activated gene 203	1.07	1.65
Asb10	ankyrin repeat and SOCS box-containing 10	0.97	1.63
Sla	src-like adaptor	1.02	1.53
Mvd	mevalonate (diphospho) decarboxylase	1.05	1.57
Amz1	archaelysin family metallopeptidase 1	0.97	1.55
Slc29a3	solute carrier family 29 (nucleoside transporters), member 3	0.94	1.54
Tmem86a	transmembrane protein 86A	0.93	1.58
Klf3	Kruppel-like factor 3 (basic)	1.01	1.57
Itgb5	integrin beta 5	0.96	1.65
Rpl22	ribosomal protein L22	0.99	1.56
Frmd4b	FERM domain containing 4B	1.18	1.54
Rassf2	Ras association (RalGDS/AF-6) domain family member 2	1.01	1.52
Tcf4	transcription factor 4	1.02	1.55
Camk1	calcium/calmodulin-dependent protein kinase I	0.95	1.52
Il18	interleukin 18	1.01	1.52
Enc1	ectodermal-neural cortex 1	1.14	1.53
Pnrc1	proline-rich nuclear receptor coactivator 1	0.90	1.57

(Continued)

Gene Symbol	Gene Name	1h	24h
Gm19773	predicted gene, 19773	0.95	1.53
Cp	ceruloplasmin	0.95	1.53
Tbc1d16	TBC1 domain family, member 16	1.00	1.58
C1qb	complement component 1, q subcomponent, beta polypeptide	1.03	1.60

The values indicate the average of results obtained from independent experiments performed in duplicate or triplicate.
