# Supplementary material for: Ethanol Extract of Aurantiochytrium mangrovei 18W-13a Strain Possesses Anti-inflammatory Effects on Murine Macrophage RAW264 Cells
Source: Front Physiol. 2018 Sep 26;9:1205. doi: 10.3389/fphys.2018.01205 (PMC6168648; doi:10.3389/fphys.2018.01205)
Supplement: Supplementary file 3 [file Table_3.pdf]

Supplementary table 3. Genes whose expression was more than 1.5 times lower than that in the control group, following treatment with the AM18W-13a extract for 24 h.

Gene Symbol	Gene Name	Ratio	
		1h	24h
Afp	alpha fetoprotein	1.01	0.29
Treml4	triggering receptor expressed on myeloid cells-like 4	0.99	0.19
Hmox1	heme oxygenase (decycling) 1	1.34	0.31
Gsta3	glutathione S-transferase, alpha 3	1.13	0.40
Ptgs2	prostaglandin-endoperoxide synthase 2	1.12	0.39
Slc40a1	solute carrier family 40 (iron-regulated transporter), member 1	0.99	0.26
Slc7a11	solute carrier family 7 (cationic amino acid transporter, y+ system), member 11	0.88	0.30
Blvrb	biliverdin reductase B (flavin reductase (NADPH))	0.93	0.29
Slc7a1	solute carrier family 7 (cationic amino acid transporter, y+ system), member 1	0.93	0.49
Gclm	glutamate-cysteine ligase, modifier subunit	1.01	0.27
Akr1b8	aldo-keto reductase family 1, member B8	1.02	0.28
Fcrl1	Fc receptor-like 1	0.97	0.40
Adh7	alcohol dehydrogenase 7 (class IV), mu or sigma polypeptide	1.00	0.37
Procr	protein C receptor, endothelial	0.87	0.32
Ptgir	prostaglandin I receptor (IP)	0.91	0.44
Srxn1	sulfiredoxin 1 homolog (S. cerevisiae)	0.95	0.40

(Continued)

Gene Symbol	Gene Name	Ratio	
		1h	24h
Ccl3	chemokine (C-C motif) ligand 3	1.12	0.34
Pcx	pyruvate carboxylase	1.00	0.52
Layn	layilin	0.98	0.43
Clec4n	C-type lectin domain family 4, member n	0.92	0.41
Dusp4	dual specificity phosphatase 4	0.92	0.39
Dnmt3l	DNA (cytosine-5-)-methyltransferase 3-like	1.13	0.39
Gbe1	glucan (1,4-alpha-), branching enzyme 1	1.12	0.40
Nrp1	neuropilin 1	0.98	0.45
Plk2	polo-like kinase 2	1.01	0.39
Gstp1	glutathione S-transferase, pi 1	1.01	0.46
Hs3st3b1	heparan sulfate (glucosamine) 3-O-sulfotransferase 3B1	1.12	0.44
Cd36	CD36 antigen	0.98	0.41
Gsr	glutathione reductase	0.95	0.46
Dcstamp	dentrocyte expressed seven transmembrane protein	1.05	0.46
Serpinb1b	serine (or cysteine) peptidase inhibitor, clade B, member 1b	1.07	0.51
Rnf128	ring finger protein 128	0.97	0.50
Serpinb9	serine (or cysteine) peptidase inhibitor, clade B, member 9	1.03	0.47

(Continued)

Gene Symbol	Gene Name	Ratio	
		1h	24h
Cat	catalase	0.92	0.51
Mfap3l	microfibrillar-associated protein 3-like	1.11	0.52
Serpine1	serine (or cysteine) peptidase inhibitor, clade E, member 1	1.14	0.44
Esd	esterase D/formylglutathione hydrolase	0.91	0.47
Glrx	glutaredoxin	0.91	0.53
Prkar2b	protein kinase, cAMP dependent regulatory, type II beta	1.02	0.52
Slc11a1	solute carrier family 11 (proton-coupled divalent metal ion transporters), membe	0.90	0.47
Flrt2	fibronectin leucine rich transmembrane protein 2	1.04	0.47
Ccl4	chemokine (C-C motif) ligand 4	1.12	0.43
Fosl1	fos-like antigen 1	1.11	0.43
Fkbp11	FK506 binding protein 11	1.04	0.48
Slc48a1	solute carrier family 48 (heme transporter), member 1	1.01	0.50
Ehd1	EH-domain containing 1	0.92	0.55
Plaur	plasminogen activator, urokinase receptor	0.92	0.46
Dmwd	dystrophia myotonica-containing WD repeat motif	0.91	0.51
Lpl	lipoprotein lipase	0.87	0.48
Jag1	jagged 1	0.91	0.48

(Continued)

Gene Symbol	Gene Name	Ratio	
		1h	24h
Tfrc	transferrin receptor	0.97	0.53
Msantd3	Myb/SANT-like DNA-binding domain containing 3	0.89	0.56
Slpi	secretory leukocyte peptidase inhibitor	1.01	0.52
Trem3	triggering receptor expressed on myeloid cells 3	0.97	0.57
Gfpt1	glutamine fructose-6-phosphate transaminase 1	1.05	0.51
Txnrd1	thioredoxin reductase 1	0.95	0.56
Creg1	cellular repressor of E1A-stimulated genes 1	0.92	0.55
Flt1	FMS-like tyrosine kinase 1	1.03	0.54
Igf2bp2	insulin-like growth factor 2 mRNA binding protein 2	1.00	0.50
S100a8	S100 calcium binding protein A8 (calgranulin A)	1.06	0.53
Plau	plasminogen activator, urokinase	0.99	0.53
Msr1	macrophage scavenger receptor 1	0.81	0.58
Pip5k1b	phosphatidylinositol-4-phosphate 5-kinase, type 1 beta	0.98	0.58
Gch1	GTP cyclohydrolase 1	1.02	0.54
Hmga2	high mobility group AT-hook 2	0.97	0.54
Slc16a3	solute carrier family 16 (monocarboxylic acid transporters), member 3	1.12	0.58
Abcc1	ATP-binding cassette, sub-family C (CFTR/MRP), member 1	1.08	0.54

(Continued)

Gene Symbol	Gene Name	Ratio	
		1h	24h
Cdr2	cerebellar degeneration-related 2	1.02	0.57
Ninj1	ninjurin 1	0.83	0.56
Tubb2a	tubulin, beta 2A class IIA	0.97	0.57
Pdgfb	platelet derived growth factor, B polypeptide	1.12	0.53
Phlda1	pleckstrin homology-like domain, family A, member 1	1.04	0.56
Spink5	serine peptidase inhibitor, Kazal type 5	0.94	0.47
Tmem26	transmembrane protein 26	1.10	0.51
Gpr84	G protein-coupled receptor 84	0.77	0.53
Pcdh7	protocadherin 7	1.01	0.56
Irg1	immunoresponsive gene 1	0.94	0.48
Lrp8	low density lipoprotein receptor-related protein 8, apolipoprotein e receptor	0.90	0.57
Slc2a1	solute carrier family 2 (facilitated glucose transporter), member 1	1.03	0.58
Aldh1l2	aldehyde dehydrogenase 1 family, member L2	1.23	0.54
Tnf	tumor necrosis factor	0.68	0.56
Ppap2a	phosphatidic acid phosphatase type 2A	0.84	0.54
Cbr3	carbonyl reductase 3	0.92	0.59

(Continued)

Gene Symbol	Gene Name	Ratio	
		1h	24h
Mthfd2	methylenetetrahydrofolate dehydrogenase (NAD+ dependent), methenyltetrahydrofola	1.00	0.54
Rgs1	regulator of G-protein signaling 1	1.70	0.51
Pdlim7	PDZ and LIM domain 7	1.09	0.59
Creld2	cysteine-rich with EGF-like domains 2	0.82	0.56

The values indicate the average of results obtained from independent experiments performed in duplicate or triplicate.
