# Supplementary material for: Ethanol Extract of Aurantiochytrium mangrovei 18W-13a Strain Possesses Anti-inflammatory Effects on Murine Macrophage RAW264 Cells
Source: Front Physiol. 2018 Sep 26;9:1205. doi: 10.3389/fphys.2018.01205 (PMC6168648; doi:10.3389/fphys.2018.01205)
Supplement: Supplementary file 4 [file Table_4.pdf]

Supplementary table 4. Annotation of selected genes whose expression was more than 1.5 times lower than that in the control group, following treatment with the AM18W-13a extract for 24 h.

| | Gene Symbol | Gene Name | Gene Ontology |
|---|---|---|---|
| Cluster1 (Chemotaxis) | *Abcc1* | ATP-binding cassette, sub-family C (CFTR/MRP), member 1 | Cell chemotaxis, response to oxidative stress |
| | *Ccl3* | chemokine (C-C motif) ligand 3 | monocyte chemotaxis, inflammatory response, lipopolysaccharide-mediated signaling pathway |
| | *Ccl4* | chemokine (C-C motif) ligand 4 | monocyte chemotaxis, inflammatory response, lipopolysaccharide-mediated signaling pathway |
| | *Flt1* | FMS-like tyrosine kinase 1 | Angiogenesis, monocyte chemotaxis |
| | *Nrp1* | neuropilin 1 | Angiogenesis, positive chemotaxis, response to wounding |
| | *Pdgfb* | platelet derived growth factor, B polypeptide | monocyte chemotaxis, substrate-dependent cell migration |
| | *Plau* | plasminogen activator, urokinase | positive regulation of cell migration, wound healing, NF-kappa B signaling pathway |
| | *S100a8* | S100 calcium binding protein A8 (calgranulin A) | immune system process, chemotaxis leukocyte migration involved in inflammatory response |

(Continued)

| | Gene Symbol | Gene Name | Gene Ontology |
|---|---|---|---|
| Cluster 5 (Inflammatory response) | *Irg1* | immunoresponsive gene 1 | immune system process, inflammatory response, response to lipopolysaccharide |
| | *Serpine1* | serine (or cysteine) peptidase inhibitor, clade E, member 1 | positive regulation of inflammatory response, cellular response to lipopolysaccharide, wound healing |
| | *Tnf* | tumor necrosis factor | IL-10 Anti-inflammatory Signaling Pathway, response to lipopolysaccharide |
| | *Trem3* | triggering receptor expressed on myeloid cells 3 | neutrophil chemotaxis, lipopolysaccharide binding |
| Cluster 9 (Cytokine-cytokine receptor interaction) | *Cd36* | CD36 antigen | positive regulation of interleukin-6 production, cellular response to lipopolysaccharide |
| | *Clec4n* | C-type lectin domain family 4, member n | adaptive immune response |
| | *Dcstamp* | dentrocyte expressed seven transmembrane protein | immune system process, cellular response to interleukin-4 |
| | *Serpinb9* | serine (or cysteine) peptidase inhibitor, clade B, member 9 | acute inflammatory response to antigenic stimulus, T cell mediated cytotoxicity |
| | *Slc11a1* | solute carrier family 11 (proton-coupled divalent metal ion transporters), membe | cellular response to lipopolysaccharide, wound healing |
| | *Slpi* | secretory leukocyte peptidase inhibitor | Immune response response to lipopolysaccharide |

(Continued)

| | Gene Symbol | Gene Name | Gene Ontology |
|---|---|---|---|
| Others | *Gch1* | GTP cyclohydrolase 1 | response to lipopolysaccharide, response to tumor necrosis factor |
| | *Gstp1* | glutathione S-transferase, pi 1 | cellular response to lipopolysaccharide, response to tumor necrosis factor |
| | *Hmox1* | heme oxygenase (decycling) 1 | IL-10 Anti-inflammatory Signaling Pathway, wound healing involved in inflammatory response |
| | *Ptgir* | prostaglandin I receptor (IP) | inflammatory response, response to lipopolysaccharide |
| | *Ptgs2* | prostaglandin-endoperoxide synthase 2 | inflammatory response, response to lipopolysaccharide |
| | *Prkar2b* | protein kinase, cAMP dependent regulatory, type II beta | cAMP dependent regulatory, Nitric Oxide Signaling Pathway |
