# Supplementary material for: Ethanol Extract of Aurantiochytrium mangrovei 18W-13a Strain Possesses Anti-inflammatory Effects on Murine Macrophage RAW264 Cells
Source: Front Physiol. 2018 Sep 26;9:1205. doi: 10.3389/fphys.2018.01205 (PMC6168648; doi:10.3389/fphys.2018.01205)
Supplement: Supplementary file 5 [file Table_5.pdf]

Supplementary table 5. Annotation of selected genes whose expression was more than 1.5 times higher than that in the control group, following treatment with the AM18W-13a extract for 24 h.

	Gene Symbol	Gene Name	Gene Ontology
Cluster1 (Chemotaxis)	*Aif1*	allograft inflammatory factor 1	inflammatory response, positive regulation of monocyte chemotaxis
	Cxcl16	chemokine (C-X-C motif) ligand 16	receptor-mediated endocytosis, T cell chemotaxis, response to lipopolysaccharide
	Cxcr4	chemokine (C-X-C motif) receptor 4	regulation of chemotaxis
	C3ar1	complement component 3a receptor 1	complement receptor mediated signaling pathway, chemotaxis, inflammatory response
	Lgals9	lectin, galactose binding, soluble 9	immune system process, chemotaxis, response to lipopolysaccharide
	S1pr1	sphingosine-1-phosphate receptor 1	leukocyte chemotaxis, positive regulation of positive chemotaxis
	Tgfbr1	transforming growth factor, beta receptor I	NFkB activation by Nontypeable Hemophilus influenzae
	Gas6	growth arrest specific 6	positive regulation of cytokine-mediated signaling pathway, negative regulation of interleukin-6 production

(Continued)

	Gene Symbol	Gene Name	Gene Ontology
Cluster 5 (Inflammatory response)	*Clec7a*	C-type lectin domain family 7, member a	leukocyte activation involved in immune response, inflammatory response
	Fcgr3	Fc receptor, IgG, low affinity III	neutrophil chemotaxis, regulation of immune response
Cluster 9 (Cytokine-cytokine receptor interaction)	*Blnk*	B cell linker	immune response
	Cd28	CD28 antigen	immune response, positive regulation of interleukin-10 production,
	Ifi44l	interferon-induced protein 44	immune response, Jak-STAT signaling pathway
	Il6ra	interleukin 6 receptor, alpha	positive regulation of interleukin-6 production, positive regulation of JAK-STAT cascade
	Il10ra	interleukin 10 receptor, alpha	:IL-10 Anti-inflammatory Signaling Pathway, interleukin-10 receptor activity,
	Il18	interleukin 18	:IL12 and Stat4 Dependent Signaling Pathway in Th1 Development, lipopolysaccharide-mediated signaling pathway

(Continued)

	Gene Symbol	Gene Name	Gene Ontology
Cluster 17 (Immunity)	*C1qb*	complement component 1, q subcomponent, beta polypeptide	innate immune response
Others	*Id3*	inhibitor of DNA binding 3	response to wounding
	Irf9	interferon regulatory factor 9	IFN alpha signaling pathway, Jak-STAT signaling pathway
	Lyst	lysosomal trafficking regulator	neutrophil mediated immunity, eukocyte chemotaxis
	St6gal1	beta galactoside alpha 2,6 sialyltransferase 1	negative regulation of chemotaxis
	Tifab	TRAF-interacting protein with forkhead-associated domain, family member B	I-kappaB kinase/NF-kappaB signaling,
	Tsc22d3	TSC22 domain family, member 3	negative regulation of activation-induced cell death of T cells, AP1-binding
